# Mechanisms and consequences of hepatic regulation of mTORC1 by metformin

**DOI:** 10.1186/2049-3002-2-S1-P28

**Published:** 2014-05-28

**Authors:** Jessica J Howell, Kristina Hellberg, Reuben J Shaw, Brendan D Manning

**Affiliations:** 1 Department of Genetics and Complex Diseases, Harvard School of Public Health, Boston, MA, USA; 2 Howard Hughes Medical Institute, Molecular and Cell Biology Laboratory, The Salk Institute for Biological Studies, La Jolla, CA, USA

## Background

In mammals, the ability to sense and respond to both intracellular and extracellular nutrient levels requires the integration and cooperation of multiple complex metabolic regulatory networks. Key among these are the mTOR and AMPK signaling pathways, which are activated in response to increased or decreased cellular energy levels, respectively. These pathways control cell growth, proliferation, metabolism and autophagy, and their dysregulation has been associated with both cancer and metabolic disease. The anti-diabetes drug metformin has been shown to function, at least in part, through activation of AMPK, however it also inhibits mTORC1 signaling, suggesting a potential role for mTORC1 inhibition in the beneficial effects of metformin. AMPK can suppress mTORC1 through activation of the TSC complex, an inhibitor of mTORC1, or inhibition of Raptor, an essential component of mTORC1. The liver plays a critical role in the maintenance of systemic metabolic homeostasis, and is thought to be the major target organ for the physiological effects of metformin, thus we are investigating the hepatic regulation of mTORC1 by metformin.

## Materials and methods

The effects of metformin on mTORC1 were assessed in livers and primary hepatocytes from LTSC1KO mice that have conditional loss of TSC1 in the liver. mTORC1 activity was determined by analysis of the phosphorylation state of its targets, and the rate of protein synthesis using 35S-methionine incorporation. Physiological effects of metformin were determined using glucose and metformin tolerance tests.

## Results

Dose-response and time-course assessments of mTORC1 activity in response to metformin show that inhibition of mTORC1 signaling is resistant to metformin in primary hepatocytes of LTSC1KO mice under conditions that fully suppress mTORC1 signaling in control hepatocytes. The rate of protein synthesis is also maintained at a higher level in these hepatocytes. Furthermore, while metformin results in decreased phosphorylation of mTORC1 targets in the livers of control mice, its activation is not as severely compromised in livers of LTSC1KO mice. However, conditional loss of TSC1 in the liver seems to have no effect on the systemic glucose lowering ability of metformin.

## Conclusions

Our findings demonstrate that conditional disruption of the TSC1-TSC2 complex desensitizes hepatocytes to the inhibition of mTORC1 by metformin, suggesting a dominant role for this tumor suppressor complex in the hepatic control of mTORC1 by energy stress. We are continuing to use this genetic model to determine the contribution of mTORC1 inhibition by metformin in the liver to the cellular and physiological effects of metformin.

